# Genome Sequence of *Salmonella enterica* Serotype Tennessee Strain CDC07-0191, Implicated in the 2006-2007 Multistate Food-Borne Outbreak Linked to Peanut Butter in the United States

**DOI:** 10.1128/genomeA.00260-13

**Published:** 2013-05-23

**Authors:** Xiangyu Deng, Joelle K. Salazar, Stephanie Frezet, Duncan MacCannell, Efrain M. Ribot, Patricia I. Fields, W. Florian Fricke, Wei Zhang

**Affiliations:** National Center for Emerging and Zoonotic Infectious Diseases, Centers for Disease Control and Prevention, Atlanta, Georgia, USAa; Institute for Food Safety and Health, Illinois Institute of Technology, Bedford Park, Illinois, USAb; Beckman Coulter Genomics, Danvers, Massachusetts, USAc; Institute for Genome Sciences, University of Maryland, School of Medicine, Department of Microbiology and Immunology, Baltimore, Maryland, USAd

## Abstract

*Salmonella enterica* serotype Tennessee strain CDC07-0191 was isolated from the 2006-2007 multistate food-borne outbreak linked to peanut butter in the United States. Here we report a high-quality draft assembly of the genome sequence of this strain, derived from a patient. This is the first reported high-quality draft genome sequence for *S. enterica* serotype Tennessee, which will enable in-depth studies of its transmission and virulence.

## GENOME ANNOUNCEMENT

*Salmonella enterica* is a frequent food contaminant and the leading cause of food-borne bacterial illnesses in the United States (1). Between August 2006 and February 2007, *S. enterica* serotype Tennessee was implicated in a major food-borne disease outbreak linked to contaminated peanut butter. A case-control study identified a total of 715 cases from 48 states, including 93 hospitalizations (2). This was the first reported outbreak of salmonellosis linked to peanut butter in the United States.

Cases of salmonellosis caused by *S. enterica* serotype Tennessee (6,7,z29:−) are relatively infrequent, with this serotype accounting for approximately 0.1% of all *Salmonella* serotypes annually reported to the National *Salmonella* Surveillance System from 1995 to 2009 (3). Compared with other *S. enterica* serotypes, Tennessee appeared to be more frequently associated with urinary tract infections (4). The strain CDC07-0191 was recovered from the urine sample of an epidemiologically linked patient in the 2006-2007 outbreak linked to peanut butter. An earlier draft genome sequence of this strain was first produced by 454 technologies and is available at http://www.ncbi.nlm.nih.gov/Traces/wgs/?val=ACBF01.

*S. enterica* Tennessee CDC07-0191 was cultured in Luria-Bertani broth at 37°C and grown to stationary phase. Genomic DNA was prepared using the ArchivePure DNA cell/tissue kit (5 PRIME Inc., Gaithersburg, MD) per the manufacturer’s instructions. Whole-genome sequencing entailed a combination of GS FLX and Illumina paired-end sequencing technologies (Roche Life Science, Mannheim, Germany; Illumina Inc., San Diego, CA) and PCR-Sanger finishing reactions; both strategies were performed at Beckman Coulter Genomics, Danvers, MA. For the GS-FLX sequencing, one fragment library and one mate-pair library containing 4-kb inserts were constructed. Totals of 150,000 and 225,000 reads were produced from these 2 libraries, respectively, corresponding to a 30-fold coverage of the genome. Approximately 2.5 million single-ended reads of 100 bp were used from an Illumina HiSeq 2000 lane to reach 50-fold coverage. A hybrid *de novo* assembly using 454 and Illumina reads simultaneously was then performed with the Mira assembler (5). Scaffolding was performed with Consed (6), and *in silico* curation was performed to join 62 contigs. The final order and orientation of the resulting contigs were analyzed using the Mauve aligner (7) and confirmed by PCR. Primer walking was performed for gap closure using Consed. The final assembly comprises one circular chromosome with a size of 4.84 Mb. Seven rRNA operons were identified in this genome; four remaining gaps of 1.8 kb, 3 kb, 0.8 kb, and 2 kb were all rRNA operons.

The availability of the high-quality draft genome sequence of *S. enterica* serotype Tennessee will enable more in-depth studies of the food-borne transmission and virulence mechanisms of this pathogen that caused human infections. Comparative analyses of this genome with other *S. enterica* genomes will shed new light on the evolutionary history responsible for the emergence of this serotype as a cause of epidemic food-borne outbreaks.

### Nucleotide sequence accession numbers.

This whole-genome shotgun project has been deposited at DDBJ/EMBL/GenBank under the accession no. APWL00000000. The version described in this paper is the first version, APWL01000000.
